# Burden of cardiovascular risk factors and disease among patients with type 1 diabetes: results of the Australian National Diabetes Audit (ANDA)

**DOI:** 10.1186/s12933-018-0726-8

**Published:** 2018-06-02

**Authors:** Anthony Pease, Arul Earnest, Sanjeeva Ranasinha, Natalie Nanayakkara, Danny Liew, Natalie Wischer, Sofianos Andrikopoulos, Sophia Zoungas

**Affiliations:** 1 School of Public Health and Preventive Medicine, Monash University, 5th Floor, The Alfred Centre, 99 Commercial Road, Melbourne, VIC 3004 Australia; 2 Diabetes and Vascular Medicine Unit, Monash Health, Clayton, VIC 3168 Australia; 3 Department of Medicine, The University of Melbourne, Melbourne, VIC 3010 Australia

**Keywords:** Type 1 diabetes mellitus, Cardiovascular disease, Epidemiology

## Abstract

**Background:**

Cardiovascular risk stratification is complex in type 1 diabetes. We hypothesised that traditional and diabetes-specific cardiovascular risk factors were prevalent and strongly associated with cardiovascular disease (CVD) among adults with type 1 diabetes attending Australian diabetes centres.

**Methods:**

De-identified, prospectively collected data from patients with type 1 diabetes aged ≥ 18 years in the 2015 Australian National Diabetes Audit were analysed. The burden of cardiovascular risk factors [age, sex, diabetes duration, glycated haemoglobin (HbA1c), blood pressure, lipid profile, body mass index, smoking status, retinopathy, renal function and albuminuria] and associations with CVD inclusive of stroke, myocardial infarction, coronary artery bypass graft surgery/angioplasty and peripheral vascular disease were assessed. Restricted cubic splines assessed for non-linearity of diabetes duration and likelihood ratio test assessed for interactions between age, diabetes duration, centre type and cardiovascular outcomes of interest. Discriminatory ability of multivariable models were assessed with area under the receiver operating characteristic (ROC) curves.

**Results:**

Data from 1169 patients were analysed. Mean (± SD) age and median diabetes duration was 40.0 (± 16.7) and 16.0 (8.0–27.0) years respectively. Cardiovascular risk factors were prevalent including hypertension (21.9%), dyslipidaemia (89.4%), overweight/obesity (56.4%), ever smoking (38.5%), albuminuria (31.1%), estimated glomerular filtration rate < 60 mL/min/1.73 m^2^ (10.3%) and HbA1c > 7.0% (53 mmol/mol) (81.0%). Older age, longer diabetes duration, smoking and antihypertensive therapy use were positively associated with CVD, while high density lipoprotein-cholesterol and diastolic blood pressure were negatively associated (p < 0.05). Association with CVD and diabetes duration remained constant until 20 years when a linear increase was noted. Longer diabetes duration also had the highest population attributable risk of 6.5% (95% CI 1.4, 11.6). Further, the models for CVD demonstrated good discriminatory ability (area under the ROC curve 0.88; 95% CI 0.84, 0.92).

**Conclusions:**

Cardiovascular risk factors were prevalent and strongly associated with CVD among adults with type 1 diabetes attending Australian diabetes centres. Given the approximate J-shaped association between type 1 diabetes duration and CVD, the impact of cardiovascular risk stratification and management before and after 20 years duration needs to be further assessed longitudinally. Diabetes specific cardiovascular risk stratification tools incorporating diabetes duration should be an important consideration in future guideline development.

**Electronic supplementary material:**

The online version of this article (10.1186/s12933-018-0726-8) contains supplementary material, which is available to authorized users.

## Background

Cardiovascular disease (CVD) is the leading cause of death among people with type 1 diabetes [1–6]. Furthermore, people with type 1 diabetes experience cardiovascular events about 10 years earlier than a matched population without diabetes [7]. This is juxtaposed with current national strategies in primary prevention of CVD that focus on absolute cardiovascular risk stratification from around 40 years of age regardless of comorbidities [1, 8–11]. This strategy fails to integrate the duration of exposure to risk factors which may be of particular relevance to younger people diagnosed with type 1 diabetes in their youth.

While traditional cardiovascular risk factors are expected to contribute to the observed increased risk of CVD, the relative strength of associations in type 1 diabetes is not clear. The protective association of female sex with CVD, for example, appears to be negated in at least those women aged less than 40 years with type 1 diabetes [5, 12, 13]. Similarly, while obesity is recognised as an independent risk factor for CVD in the general population [14–18], the impact of increasing body mass index (BMI) in type 1 diabetes is not firmly established. Furthermore, recommendations for pharmacotherapy to manage risk factors are largely extrapolated from trials in adults with type 2 diabetes that may not be generalisable to those with type 1 diabetes [1, 8, 9, 19].

Understanding relationships between risk factors and cardiovascular outcomes is pivotal for informing preventive strategies. Current risk stratification models utilise traditional cardiovascular risk factors from risk equations, which have been extensively validated in the general population [1, 8–10]. However, this approach has been shown to be a poor predictor of cardiovascular events in adults with type 1 diabetes, generally underestimating risk in this group [1, 9, 20]. Risk stratification models specifically for adults with type 1 diabetes as well as investigational biomarkers have been developed but are not in widespread clinical use [21–26]. Elements of these models that differ from those currently recommended include consideration of diabetes duration, glycaemic control (HbA1c) and albuminuria [21–23]. However, there is a paucity of contemporary data on the prevalence of cardiovascular risk factors and disease among people with type 1 diabetes. Follow-up of the landmark diabetes control and complications trial cohort also suggests there may be gaps in managing cardiovascular risk factors as only 7.6% attained all four of the American Diabetes Association recommendations for complication prevention [27]. Until further studies can corroborate any associations and the reliability of new risk stratification models, only individual risk factor assessment and clinical judgment can direct clinical care.

We thus examined the burden of cardiovascular risk factors and their associations with cardiovascular complications among patients with type 1 diabetes attending diabetes centres across Australia. Traditionally considered and diabetes specific cardiovascular risk factors were hypothesised to be prevalent and strongly associated with CVD in this vulnerable population.

## Methods

### Subjects

The Australian National Diabetes Audit (ANDA) is an annual cross-sectional benchmarking activity including patients of all ages and diabetes types. Diabetes centres voluntarily participate, with approximately two-thirds being tertiary centres and one-third primary or community based centres. De-identified data for our study were collected across all centres during a 1-month survey period in May or June (2015) for all consecutive patients. Patients considered for this analysis were adults (≥ 18 years) with type 1 diabetes (n = 1169) presenting to one of the 49 participating diabetes centres. The degree of patient ascertainment could not be determined because only data for those participants involved in the study were collected.

Ethical approval for our study was provided by the Monash Health Human Research Ethics Committee.

### Data collection

Relevant pre-specified sociodemographic (date of birth, date of diabetes diagnoses, sex, Aboriginal/Torres Strait Islander ethnicity) and clinical variables (diabetes type, weight, height, smoking status, blood pressure (BP), lipid levels, urinary albumin, serum creatinine, HbA1c, lipid lowering medications, antihypertensive medications, diabetes complications, comorbid conditions) were collected. Health care professionals participating in ANDA examined patients, reviewed medical records including pathology results during standard patient consultations and recorded the de-identified information in a standardised collection form (Additional file 1). The participating centres were later contacted to clarify missing data and invalid entries.

### Variables

Age was calculated as the date of questionnaire (in 2015) minus the date of birth, and diabetes duration was calculated as the date of questionnaire minus the date of diabetes diagnosis. Provided height and weight measurements were used to calculate the BMI in kg/m^2^. The main outcome variables for this analysis were cerebral stroke, myocardial infarction (MI), coronary artery bypass graft (CABG) surgery/angioplasty, peripheral vascular disease (PVD) and the composite of these atherosclerotic outcomes defining CVD. PVD was defined clinically as the absence of both the dorsalis pedis and posterior tibialis pulses on either foot or amputation of toe, forefoot or leg (above or below knee), not due to trauma or causes other than vascular disease. An additional outcome of interest was congestive cardiac failure (CCF) defined by clinician determined symptomatic status and responsiveness to therapy. The healthcare professional completing the questionnaire determined the presence of these complications and other comorbid conditions with access to a data dictionary of terms provided by the ANDA secretariat prior to commencing the questionnaire (Additional file 2). Cardiovascular risk factors considered in analysis include sex, age, diabetes duration, HbA1c, BMI categories, smoking status (ever smoked versus never smoked), systolic blood pressure, diastolic blood pressure, albuminuria (> 20.0 mg/L, > 20.0 μg/min, > 30.0 mg/24 h, or > 2.5 mg/mmol for women and > 3.5 mg/mmol for men), presence of retinopathy, high density lipoprotein-cholesterol (HDL-C) and estimated glomerular filtration rate (eGFR) calculated using the chronic kidney disease epidemiology collaboration (CKD-EPI) formula based on sex and collected creatinine values in μmol/L [28]. The eGFR was not adjusted for ethnicity among Aboriginal or Torres Strait Islander (ATSI) people groups in keeping with current literature [29–31], and no other ethnicity data was collected. Total cholesterol, low density lipoprotein-cholesterol (LDL-C) and triglycerides were excluded from regression analyses a priori. BMI categories were considered as underweight (< 18.5 kg/m^2^), normal weight (18.5 to < 25.0 kg/m^2^), overweight (25.0 to < 30.0 kg/m^2^) and obese (≥ 30.0 kg/m^2^). Dyslipidaemia was defined as failure to meet current Australian treatment targets (i.e. total cholesterol ≥ 4.0 mmol/L, HDL-C < 1.0 mmol/L, LDL-C ≥ 2.0 mmol/L or triglycerides ≥ 2.0 mmol/L). Hypertension was defined as systolic blood pressure ≥ 140 mmHg or diastolic blood pressure ≥ 90 mmHg. Retinopathy was recorded as absent or present for the preceding 12 months. Diabetes centre type corresponds to secondary or community/primary centres derived from the category of membership with the National Association of Diabetes Centres (NADC). Secondary centres comprised centres of excellence and tertiary diabetes centres, and community/primary centres comprise affiliate and diabetes care centres.

### Statistical analysis

Continuous data were tested for normality of distribution and summarised as means with standard deviations (± SD) or medians with interquartile range (IQR; 25th–75th percentile). When comparing means or medians we used the Student’s *t* test or Mann–Whitney U test respectively. Categorical variables were summarised as participant numbers and percentages, and when comparing between groups we used the Chi square test. Restricted cubic splines were utilised to evaluate non-linear associations between cardiovascular outcomes and diabetes duration. Scoping review and expert opinion lead to selection of knots at 5.0, 15.0, 25.0 and 35.0 years duration. The binary logistic regression model was used to examine the association of risk factors with cardiovascular outcomes of interest and likelihood ratio test assessed for interactions between age and diabetes duration as well as diabetes centre type. The selection of variables was based on identifying all measured clinical variables of known or suspected prognostic importance for the outcomes of interest and/or exhibiting a p value ≤ 0.1 on univariable analysis. Age and sex were forced into all multivariable models as they were considered clinically significant a priori. Models also adjusted for antihypertensive and lipid lowering therapy. Multivariable regression analyses were performed for each cardiovascular outcome of interest using stepwise selection of variables (1% probability for entry and 5% probability for removal) for the remaining predictor variables. Based on the coefficients from the final parsimonious multivariable model, we calculated the ROC curve and 95% confidence intervals. Population attributable risk (PAR) and 95% confidence intervals were calculated for each significant categorical variable from the final multivariable models under the assumption that associations were causal [32]. Multiple imputation was performed for missing data (Additional file 3: Table S1 and Additional file 4: Table S2 respectively). Statistical analyses were performed using Stata software version 14.2 (StataCorp, Texas, USA) and level of significance set at 5% unless otherwise specified.

## Results

### Patient characteristics

Data from 1169 patients were included in this study. Cardiovascular risk factors were highly prevalent, including hypertension (21.9%), dyslipidaemia (89.4%), overweight or obesity (56.4%), ever smoking (38.5%), albuminuria (31.1%), eGFR < 45 mL/min/1.73 m^2^ (6.5%) or < 60 mL/min/1.73 m^2^ (10.3%) and HbA1c exceeding 7.0% (81.0%). Patients with CVD tended to be male (61.5%) with a mean age of 58.5 ± 13.7 years. Median diabetes duration was 35.0 (24.5–45.0) years, mean HbA1c was 8.6 ± 1.5% and the mean HDL-C was 1.35 ± 0.42 mmol/L. Most patients with CVD were overweight/obese (56.4%), had smoked (64.2%) or had retinopathy (56.2%). The mean eGFR for patients with CVD was 71 (± 29) mL/min/1.73 m^2^ and around half of the patients with CVD had albuminuria (47.9%). Secondary prevention prescribing of lipid lowering therapy and antihypertensive therapy was noted in up to 75.3 and 72.9% respectively. Mean systolic and diastolic blood pressure levels were 132 ± 21 and 72 ± 10 mmHg respectively. A summary of cardiovascular outcomes with risk factor levels is provided in Table 1 and Additional file 5: Table S3a and b.

**Table 1:** Distribution of variables for cardiovascular outcomes of interest

Variables	Cardiovascular outcomes of interest			p-value
	Total N = 1169	CVD N = 148	No CVD N = 1013	
Female sex, n (%)	609 (53.3%)	57 (38.5%)	550 (55.7%)	< 0.001
Age (years), mean (± SD)	40.0 (± 16.7)	58.5 (± 13.7)	37.3 (± 15.4)	< 0.001
Age (years), median (IQR)	37.0 (24.9-52.0)	59.0 (49.0-68.2)	33.8 (23.9-47.2)	< 0.001
Diabetes duration (years), mean (SD)	19.2 (± 14.4)	34.8 (± 15.5)	17.1 (± 12.8)	< 0.001
Diabetes duration (years), median (IQR)	16.0 (8.0-27.0)	35.0 (24.5-45.0)	15.0 (8.0-24.0)	< 0.001
Diabetes duration (≥ 20.0 years), n (%)	476 (41.3%)	121 (84.0%)	354 (35.4%)	< 0.001
HbA1c (%), mean (± SD)	8.5 (± 1.8)	8.6 (± 1.5)	8.5 (± 1.9)	0.386
HDL-C^a^, mean (± SD)	1.53 (± 0.54)	1.35 (± 0.42)	1.56 (± 0.56)	< 0.001
LDL-C^a^, mean (± SD)	2.55 (± 0.95)	2.15 (± 0.82)	2.62 (± 0.95)	< 0.001
Total-C^a^, mean (± SD)	4.73 (± 1.09)	4.18 (± 1.12)	4.83 (± 1.06)	< 0.001
Triglycerides^a^, mean (± SD)	1.37 (± 1.42)	1.54 (± 1.84)	1.34 (± 1.34)	0.190
Systolic BP^b^, mean (± SD)	124 (± 17)	132 (± 21)	123 (± 16)	< 0.001
Diastolic BP^b^, mean (± SD)	74 (± 10)	72 (± 10)	74 (± 10)	0.073
BMI categories, n (%) (kg/m^2^)				0.372
< 18.5	20 (2.0%)	4 (3.2%)	15 (1.7%)	
18.5 to < 25	419 (41.6%)	51 (40.5%)	365 (41.8%)	
25 to < 30	315 (31.3%)	34 (27.0%)	279 (31.9%)	
≥ 30	253 (25.1%)	37 (29.4%)	215 (24.6%)	
Ever smoked, n (%)	397 (38.5%)	86 (64.2%)	309 (34.6%)	< 0.001
Albuminuria, n (%)	220 (31.1%)	46 (47.9%)	174 (28.6%)	< 0.001
eGFR^c^, mean (± SD)	97 (± 28)	71 (± 29)	101 (± 25)	< 0.001
Antihypertensive Rx, n (%)	320 (28.1%)	105 (72.9%)	214 (21.7%)	< 0.001
Lipid lowering Rx, n (%)	342 (29.7%)	110 (75.3%)	232 (23.2%)	< 0.001
Retinopathy, n (%)	284 (24.7%)	82 (56.2%)	202 (20.2%)	< 0.001

Rx: treatment

^a^mmol/L, ^b^ mmHg, ^c^ mL/min/1.73 m^2^

### Cardiovascular complications

**Fig. 1:**
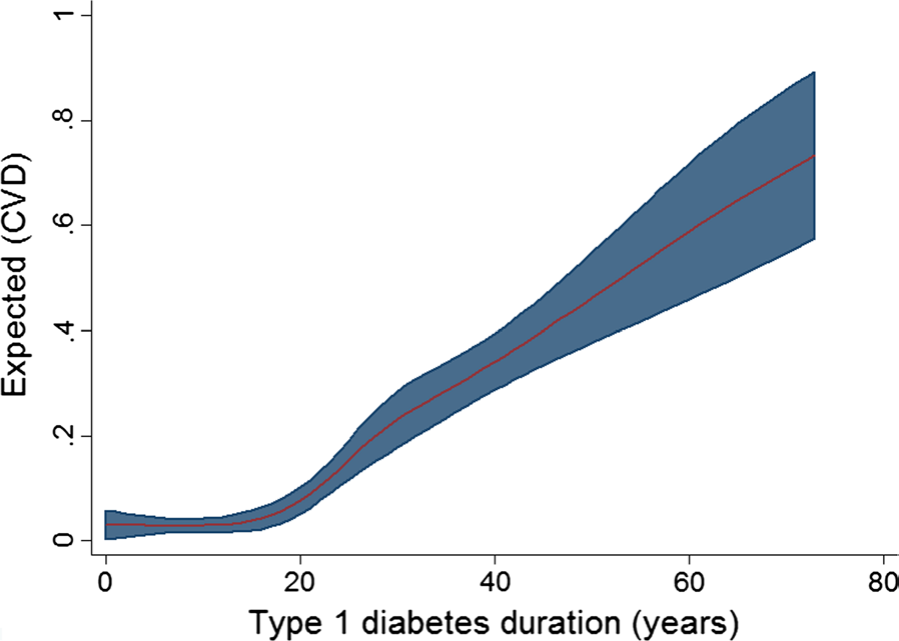
Restricted cubic spline of type 1 diabetes duration and cardiovascular disease

A non-linear association between diabetes duration and CVD was demonstrated (Fig. 1). Odds of CVD were low and static until approximately 20 years duration, at which point a positive linear association emerged (Fig. 1). As a categorical variable, diabetes duration ≥ 20.0 years was significantly associated with the composite outcome of CVD (no interaction with age; likelihood ratio test p-value 0.816) in multivariable analysis [adjusted odds ratio (aOR) 1.05 (95% CI 1.01, 1.10); p 0.018] (Table 2).

**Table 2:** Risk factors associated with cardiovascular outcomes of interest

Variables	Univariable analyses		Multivariable analyses			
	OR (95% CI)	p-value	OR (95% CI)	p-value	ROC	95% CI
Cardiovascular disease (composite)						
Female sex	0.50 (0.35–0.71)	< 0.001	0.90 (0.46–1.78)	0.764	0.88	0.84–0.92
Age (years)	1.08 (1.07–1.09)	< 0.001	1.06 (1.03–1.09)	< 0.001		
Diabetes duration group	1.12 (1.09–1.15)	< 0.001	1.05 (1.01–1.10)	0.018		
HbA1c (%)	1.04 (0.95–1.15)	0.386				
HDL-cholesterol (mmol/L)	0.36 (0.21–0.62)	< 0.001	0.43 (0.21–0.90)	0.025		
Systolic BP (mmHg)	1.03 (1.02–1.04)	< 0.001				
Diastolic BP (mmHg)	0.98 (0.97–1.00)	0.073	0.96 (0.93–1.00)	0.048		
BMI categories	1.00 (0.97–1.03)	0.937				
Ever smoked	3.39 (2.32–4.95)	< 0.001	2.40 (1.26–4.58)	0.008		
Albuminuria	2.30 (1.49–3.56)	< 0.001				
eGFR (mL/min/1.73 m^2^)	0.96 (0.96–0.97)	< 0.001				
Antihypertensive Rx	9.74 (6.54–14.49)	< 0.001	2.44 (1.15–5.18)	0.020		
Lipid lowering Rx	10.13 (6.76–15.17)	< 0.001				
Retinopathy	5.07 (3.53–7.28)	< 0.001				
Stroke						
Female sex	0.33 (0.15–0.72)	0.006	0.49 (0.16–1.47)	0.201	0.81	0.74–0.88
Age (years)	1.06 (1.04–1.09)	< 0.001	1.05 (1.01–1.08)	0.006		
Diabetes duration group	1.08 (1.03–1.12)	< 0.001				
HbA1c (%)	1.17 (0.99–1.38)	0.062				
HDL-cholesterol (mmol/L)	0.26 (0.09–0.77)	0.015				
Systolic BP (mmHg)	1.02 (1.00–1.04)	0.016				
Diastolic BP (mmHg)	1.00 (0.97–1.04)	0.928				
BMI categories	1.02 (0.96–1.10)	0.514				
Ever smoked	2.32 (1.10–4.92)	0.027				
Albuminuria	2.27 (0.97–5.31)	0.059				
eGFR (mL/min/1.73 m^2^)	0.97 (0.96–0.99)	< 0.001	0.98 (0.96–1.00)	0.030		
Antihypertensive Rx	7.85 (3.47–17.74)	< 0.001				
Lipid lowering Rx	7.54 (3.35–16.95)	< 0.001				
Retinopathy	3.87 (1.88–7.96)	< 0.001				
Myocardial infarction						
Female sex	0.41 (0.23–0.72)	0.002	0.97 (0.39–2.41)	0.943	0.90	0.87–0.94
Age (years)	1.08 (1.06–1.10)	< 0.001	1.09 (1.05–1.13)	< 0.001		
Diabetes duration group	1.12 (1.08–1.17)	< 0.001				
HbA1c (%)	0.96 (0.82–1.13)	0.647				
HDL-cholesterol (mmol/L)	0.24 (0.10–0.58)	0.002	0.20 (0.06–0.68)	0.010		
Systolic BP (mmHg)	1.03 (1.02–1.05)	< 0.001				
Diastolic BP (mmHg)	1.01 (0.98–1.03)	0.707				
BMI categories	1.01 (0.95–1.06)	0.842				
Ever smoked	2.31 (1.30–4.10)	0.004				
Albuminuria	2.20 (1.15–4.21)	0.017				
eGFR (mL/min/1.73 m^2^)	0.97 (0.96–0.98)	< 0.001				
Antihypertensive Rx	23.91 (10.12–56.49)	< 0.001	5.06 (1.38–18.54)	0.014		
Lipid lowering Rx	18.21 (8.14–40.73)	< 0.001				
Retinopathy	3.67 (2.12–6.35)	< 0.001				
Coronary artery bypass graft/angioplasty						
Female sex	0.38 (0.22–0.68)	0.001	0.93 (0.34–2.52)	0.884	0.92	0.89-0.95
Age (years)	1.09 (1.07–1.12)	< 0.001	1.08 (1.03–1.13)	0.001		
Diabetes duration group	1.21 (1.13–1.30)	< 0.001				
HbA1c (%)	0.96 (0.81–1.12)	0.586				
HDL-cholesterol (mmol/L)	0.27 (0.11–0.67)	0.005	0.23 (0.06–0.92)	0.038		
Systolic BP (mmHg)	1.03 (1.01–1.04)	< 0.001				
Diastolic BP (mmHg)	0.98 (0.96–1.01)	0.171				
BMI categories	1.03 (0.98–1.09)	0.263				
Ever smoked	2.18 (1.25–3.81)	0.006				
Albuminuria	1.82 (0.93–3.59)	0.082				
eGFR (mL/min/1.73 m^2^)	0.97 (0.96–0.98)	< 0.001				
Antihypertensive Rx	38.63 (13.83–107.88)	< 0.001	8.96 (1.12–71.54)	0.039		
Lipid lowering Rx	36.00 (12.91–100.41)	< 0.001				
Retinopathy	4.45 (2.57–7.69)	< 0.001				
Peripheral vascular disease						
Female sex	0.73 (0.46–1.16)	0.180	1.08 (0.49–2.39)	0.851	0.85	0.81–0.90
Age (years)	1.07 (1.05–1.09)	< 0.001	1.04 (1.01–1.07)	0.005		
Diabetes duration group	1.11 (1.08–1.15)	< 0.001				
HbA1c (%)	1.09 (0.96–1.24)	0.171				
HDL-cholesterol (mmol/L)	0.37 (0.18–0.77)	0.008				
Systolic BP (mmHg)	1.02 (1.01–1.04)	< 0.001				
Diastolic BP (mmHg)	0.97 (0.95–0.99)	0.009				
BMI categories	0.99 (0.95–1.03)	0.653				
Ever smoked	3.67 (2.22–6.08)	< 0.001				
Albuminuria	2.97 (1.65–5.34)	< 0.001				
eGFR (mL/min/1.73 m^2^)	0.96 (0.95–0.97)	< 0.001	0.97 (0.96–0.99)	0.002		
Antihypertensive Rx	5.26 (3.25–8.53)	< 0.001				
Lipid lowering Rx	5.22 (3.21–8.46)	< 0.001				
Retinopathy	6.41 (3.95–10.41)	< 0.001	2.47 (1.06–5.74)	0.036		
Congestive cardiac failure						
Female sex	1.00 (0.36–2.78)	0.964	1.51 (0.30–7.47)	0.614	0.90	0.84–0.95
Age (years)	1.10 (1.06–1.14)	< 0.001	1.15 (1.05–1.25)	0.002		
Diabetes duration group	1.16 (1.05–1.29)	0.004				
HbA1c (%)	0.97 (0.70–1.34)	0.858				
HDL-cholesterol (mmol/L)	1.68 (0.90–3.16)	0.104				
Systolic BP (mmHg)	1.02 (0.99–1.05)	0.157				
Diastolic BP (mmHg)	0.97 (0.92–1.02)	0.195				
BMI categories	1.20 (1.05–1.38)	0.008				
Ever smoked	1.40 (0.50–3.90)	0.515				
Albuminuria	5.30 (1.36–20.70)	0.016				
eGFR (mL/min/1.73 m^2^)	0.95 (0.94–0.97)	< 0.001				
Antihypertensive Rx	37.42 (4.90–285.81)	< 0.001				
Lipid Lowering Rx	6.70 (2.12–21.21)	0.001				
Retinopathy	20.75 (4.65–92.51)	< 0.001				

Rx: treatment

Increasing age [aOR 1.06 (95% CI 1.03, 1.09)], diabetes duration ≥ 20.0 years [aOR 1.05 (95% CI 1.01, 1.10)], smoking status [aOR 2.40 (95% CI 1.26, 4.58)] and prescription of antihypertensive therapy [aOR 2.44 (95% CI 1.15, 5.18)] were all positively associated with CVD. Increasing HDL-C and diastolic blood pressure were negatively associated with CVD [aOR 0.43 (95% CI 0.21, 0.90) and aOR 0.96 (95% CI 0.93–1.00) respectively]. The model’s discriminatory ability was demonstrated with area under the ROC curve of 0.88 (95% CI 0.84, 0.92) (Table 2).

When stroke was considered, there was significant positive association with increasing age [aOR 1.05 (95% CI 1.01, 1.08)]. Increasing eGFR was negatively associated with stroke [aOR 0.98 (95% CI 0.96, 1.00)]. The area under the ROC curve was 0.81 (95% CI 0.74, 0.88) (Table 2).

When the outcome of MI or CABG/angioplasty was considered, there were significant positive associations with increasing age [aOR 1.09 (95% CI 1.05, 1.13) or aOR 1.08 (95% CI 1.03, 1.13)] and antihypertensive therapy [aOR 5.06 (95% CI 1.38, 18.54) or aOR 8.96 (95% CI 1.12, 71.54)], and negative associations with increasing HDL-C [aOR 0.20 (95% CI 0.06, 0.68) or aOR 0.23 (95% CI 0.06, 0.92)]. The area under the ROC curve for MI was 0.90 (95% CI 0.87, 0.94) and for CABG/angioplasty, it was 0.92 (95% CI 0.89, 0.95) (Table 2).

When PVD was considered, there were significant positive associations with increasing age [aOR 1.04 (95% CI 1.01, 1.07)], retinopathy [aOR 2.47 (95% CI 1.06, 5.74)], and negative association with eGFR [aOR 0.97 (95% CI 0.96, 0.99)]. The area under the ROC curve was 0.85 (95% CI 0.81, 0.90) (Table 2).

When the additional outcome of CCF was considered, there was significant association with increasing age [aOR 1.15 (95% CI 1.05, 1.25)]. The area under the ROC curve was 0.90 (95% CI 0.84, 0.95) (Table 2).

### Sensitivity analyses

Adding diabetes centre type into the final multivariable CVD models had minimal impact on the associations. There was also no significant interaction between diabetes centre type and any atherosclerotic cardiovascular outcome. Further, excluding patients with CCF resulted in diastolic blood pressure and antihypertensive therapy being removed from the final parsimonious model for CVD (data not shown).

After multiple imputation for missing data there was an increase in the magnitude of the association between antihypertensive therapy and MI or CABG/angioplasty [aOR 15.91 (95% CI 7.65, 33.12; p < 0.001) and aOR 21.90 (95% CI 9.79, 48.99; p < 0.001) respectively] and HDL-cholesterol was no longer significantly associated with CABG/angioplasty [aOR 0.45 (95% CI 0.19, 1.08; p 0.072)] (Additional file 3: Table S1).

### Population attributable risks for factors associated with cardiovascular outcomes

In the study population, the estimated proportions of CVD attributable to diabetes duration ≥ 20 years, use of antihypertensive therapy and smoking were 6.5% (95% CI 1.4, 11.6), 5.1% (95% CI 0.9, 9.3) and 3.9% (95% CI 1.0, 6.7), respectively. The estimated proportion of PVD attributable to presence of retinopathy was 2.7% (95% CI 0.2, 5.2). The estimated proportions of MI or CABG/angioplasty attributable to use of antihypertensive therapy were 4.8% (95% CI 1.8, 7.8) and 11.2% (95% CI 5.0, 17.5), respectively (Additional file 6: Table S4).

## Discussion

This study reports for the first time the large burden of cardiovascular risk factors among patients with type 1 diabetes attending diabetes centres across Australia. Furthermore it shows that a group of traditional cardiovascular risk factors (age, sex, HDL-cholesterol level, smoking status, diastolic blood pressure and use of anti-hypertensive therapy) and diabetes specific risk factors (type 1 diabetes duration), provide good discriminatory ability for the presence of CVD. The individual outcomes of MI, CABG/angioplasty and CCF share similar associations, while stroke is also associated with declining renal function and PVD is associated with declining renal function and retinopathy. This suggests that information required for cardiovascular risk stratification among patients with type 1 diabetes may not differ substantively from other high risk populations aside from the need to consider diabetes duration.

The significant non-linear association between diabetes duration and CVD (independent of patient age) and the threshold effect seen at approximately 20 years, is an important finding and consistent with previous modelling, prospective cohort and registry studies [12, 13, 22, 33–35]. Indeed, population based cohort studies and national registry studies have all observed increased rates of CVD with longer diabetes duration. Some have also reported that CVD becomes the leading cause of death after about 20 years duration [12, 13, 22, 34, 35]. The substantive PAR related to longer diabetes duration strongly supports the assessment and management of cardiovascular risk among people with long diabetes duration irrespective of their current age and the older age thresholds recommended by current CVD guidelines.

The negative association of HDL-cholesterol with CVD, MI and CABG/angioplasty [aOR 0.43 (95% CI 0.21, 0.90); 0.20 (95% CI 0.06, 0.68) and 0.23 (95% CI 0.06, 0.92) respectively] is in keeping with current understanding of a protective role for HDL-cholesterol and HDL function [1, 36–40]. While it is unknown whether increasing HDL-cholesterol will improve cardiovascular outcomes among patients with type 1 diabetes, the importance of this lipid variable for risk stratification is consistent with data from the Framingham Heart Study and a number of meta-analyses which have also reported an inverse association with CVD in other populations [36–38, 41]. The observation that pharmacotherapies were strongly associated with CVD likely relates to secondary prevention strategies.

The lack of an independent positive association between CVD and HbA1c, systolic blood pressure, BMI, albuminuria or negative association with renal function was unexpected. In particular, our finding of no association with HbA1c conflicts with other evidence of a linear relationship between hyperglycaemia or glycaemic exposure and cardiovascular risk [33, 42–48]. This may be explained by differences in the study designs as we were unable to assess glycaemic control over time. We also noted no significant difference in glycaemic control among adults with or without cardiovascular disease (p 0.386; Table 1).

Systolic blood pressure, albuminuria and declining eGFR were significantly associated with increased risk of CVD in univariable analysis [OR 1.03 (95% CI 1.02, 1.04), OR 2.30 (95% CI 1.49, 3.56) and OR 0.96 (95% CI 0.96, 0.97) respectively], but not in the multivariable analysis, suggesting these effects were accounted for by other variables in the model. Interestingly, the mean systolic and diastolic blood pressures among all patients and among those with CVD were within or close to recommended blood pressure targets measuring 124 ± 17 and 74 ± 10 mmHg, and 132 ± 21 and 72 ± 10 mmHg respectively. Albuminuria was also noted to be prevalent in 31.1% of our cohort, affecting around half (47.9%) of the patients with a history of CVD and is consistent with international estimates of 28–52% prevalence among patients with type 1 diabetes [49, 50]. This highlights the current prioritisation of blood pressure control among diabetes centres in Australia [1, 2] as well as the importance of routine screening for renal dysfunction and albuminuria.

Our finding that 38.5% of adult patients with type 1 diabetes had been smokers is consistent with a recent report that 38% of all Australians over 14 years of age have been smokers [51]. As expected, the proportion was much higher among those patients with a history CVD (64.2%), reinforcing the need for diabetes centres to offer patients assistance with smoking cessation efforts.

While elevated BMI is recognised as an independent risk factor for CVD in the general population [14–18], this relationship is not firmly established in patients with type 1 diabetes [52] and no association was noted in our analyses. Nonetheless, the finding that 56.4% of patients with type 1 diabetes were either overweight or obese is alarming but consistent with other studies in this population that report rates as high as 78% [53–55]. In addition, we found that female sex was not independently associated with any cardiovascular outcome. This supports the premise that the protective effect of female sex on cardiovascular disease is negated among women with type 1 diabetes as reported by previous cohort and registry studies [5, 12, 13].

The association between CVD and diastolic blood pressure is complex and may be impacted by patient age, arterial stiffness, vascular resistance, endothelial dysfunction, diastolic dysfunction and antihypertensive therapy [25, 26, 56–58]. This may be of particular relevance to our heterogeneous cohort ranging from 18 to 91 years of age, including patients with CCF and those taking multiple antihypertensive agents. The observed negative association between diastolic blood pressure and CVD may also represent reverse causation [59–63]. It was thus not surprising that diastolic blood pressure was removed from the prediction model when patients with CCF were excluded.

The finding that diabetic retinopathy and declining renal function was associated with peripheral vascular disease was not surprising and may relate to shared risk factors [64–66]. In our cross-sectional study, microvascular complications such as retinopathy or nephropathy provided an indication of long term risk factor exposure, but cohort studies have suggested PVD may also predict cardiovascular outcomes and end stage kidney disease [64, 67]. Further, the negative association between renal function and stroke that we observed is in keeping with studies among the general population [68–71]. However, we found no independent association between stroke and albuminuria in contrast to prior studies [72–77].

A strength of this analysis includes the large dataset of patients with type 1 diabetes taken from a nation-wide benchmarking activity. Furthermore, participants are likely to be representative of patients attending diabetes centres throughout Australia as data were collected from every state and territory. Data were also collected for a broad range of cardiovascular risk factors and clinically significant outcomes, with consideration of non-linear associations and precision of risk prediction using area under the ROC curve. Key study limitations comprise the cross-sectional nature of data collection, possible referral bias, and the reliance on healthcare worker reports as we were unable to independently verify diagnoses, treatments or biochemistry. Also, the pre-specified clinical questionnaire in ANDA did not provide scope to differentiate those patients who were normotensive or had normal lipid profiles due to medication or if pharmacotherapy was solely part of secondary prevention strategies, and these groups may confer different degrees of cardiovascular risk. Another limitation is that albuminuria was defined by a single biochemistry result within the 12 months prior to participation in ANDA. Single false positive results or resolution of albuminuria with blockade of the renin–angiotensin–aldosterone-system therefore could not be captured by this study. The association between adiposity and CVD was assessed only with BMI, but other measures such as waist circumference may add to future studies. Finally, the calculation of PAR was based on the assumption that there was a causal relationship between the risk factors identified in our study and CVD outcomes. Despite these limitations, this study provides important data on CVD among a large population with type 1 diabetes and informs future longitudinal analyses of cardiovascular risk stratification. Our findings also suggest that future cardiovascular risk stratification models will need to examine the impact of diabetes specific risk factors for populations with type 1 diabetes using the ‘Transparent Reporting of a multivariable prediction model for Individual Prognosis or Diagnosis’ (TRIPOD) statement [78].

## Conclusions

Our study demonstrates that the adult population with type 1 diabetes attending diabetes centres bears a significant cardiovascular burden. Further, analysis reveals associations between a number of traditionally considered and diabetes specific risk factors with CVD, which together provide good discriminatory ability for presence of disease. Given the substantial population risk of CVD attributable to long diabetes duration, the impact of new cardiovascular risk stratification tools and interventions to manage risk factors before and after 20 years duration will need to be further assessed by prospective studies.

## Additional files

**Additional file 1.** ‘ANDA-AQCA 2015’ provides the questionnaire that was completed as part of the Australian Quality Clinical Audit [AQCA].

**Additional file 2.** ‘ANDA-AQCA 2015 Data Definitions’ outlines the definitions used by healthcare professionals who completed the questionnaire.

**Additional file 3: Table S1.** Multiple imputation for cardiovascular outcomes of interest.

**Additional file 4: Table S2.** Missing data for cardiovascular risk factors and outcomes of interest.

**Additional file 5: Table S3.** a Distribution of variables for cardiovascular outcomes of interest and b Distribution of variables for congestive cardiac failure.

**Additional file 6: Table S4.** Population attributable risk for cardiovascular outcomes of interest.

## Abbreviations

ANDA: Australian National Diabetes Audit
aOR: adjusted odds ratio
AQCA: Australian Quality Clinical Audit
BMI: body mass index
BP: blood pressure
CABG: coronary artery bypass graft
CCF: congestive cardiac failure
CKD-EPI: chronic kidney disease epidemiology collaboration
CVD: cardiovascular disease
eGFR: estimated glomerular filtration rate
HbA1c: glycated haemoglobin
HDL-C: high density lipoprotein-cholesterol
IQR: interquartile range (25th–75th percentile)
LDL-C: low density lipoprotein-cholesterol
HDL-C: high density lipoprotein-cholesterol
MI: myocardial infarction
NADC: National Association of Diabetes Centres
PVD: peripheral vascular disease
ROC: receiver operating characteristic
Rx: treatment
SD: standard deviation
Total-C: total-cholesterol
TRIPOD: Transparent Reporting of a multivariable prediction model for Individual Prognosis or Diagnosis
