# Supplementary material for: Burden of cardiovascular risk factors and disease among patients with type 1 diabetes: results of the Australian National Diabetes Audit (ANDA)
Source: Cardiovasc Diabetol. 2018 Jun 2;17:77. doi: 10.1186/s12933-018-0726-8 (PMC5984751; doi:10.1186/s12933-018-0726-8)
Supplement: Supplementary file 1 — Additional file 1. ‘ANDA-AQCA 2015’ provides the questionnaire that was completed as part of the Australian Quality Clinical Audit [AQCA]. [file 12933_2018_726_MOESM1_ESM.pdf]

Draft

ANDA-AQCA 2015
Australian National Diabetes Audit - Australian Quality Clinical

nadc National Association of Diabetes Centres

Section 1. Patient Demographics

Medical Record No. ☐☐☐☐☐☐☐☐☐☐☐ Centre ID ☐☐☐ Site Staff Identifier ☐☐☐☐☐

1.1 Date of birth ☐☐ / ☐☐ / ☐☐☐☐ (d d / m m / y y y y)

1.2 Sex ☐ Male ☐ Female *if FEMALE* → 1.2.1 Currently pregnant ☐ No ☐ Yes

1.3 Date of visit ☐☐ / ☐☐ / 2 0 1 5 (d d / m m / y y y y)

1.4 Initial visit ☐ No ☐ Yes

1.5 Aboriginal/Torres Strait Islander ☐ No ☐ Yes

1.6 Country of birth ☐☐☐☐☐☐☐☐☐☐☐☐☐☐☐

1.7 NDSS member ☐ No ☐ Yes

1.8 DVA patient ☐ No ☐ Yes

Section 2. Diabetes Type & Management

2.1 Date of diagnosis ☐☐ / ☐☐☐☐ (m m / y y y y)

2.2 Type of diabetes ☐ Type 1 ☐ Type 2 ☐ GDM ☐ Don't know ☐ Other

2.3 Management method ☐ Diet only ☐ Acarbose ☐ GLP1 Agonist ☐ Sulphonylurea ☐ Insulin ☐ Glitazone ☐ Metformin ☐ DPP4 Inhibitor ☐ SGLT2 ☐ Nil

if INSULIN → 2.3.1 Number of years ☐☐

2.3.2 Mode ☐ Basil ☐ MDI ☐ Pump

Section 3. Height, Weight & Smoking Status

3.1 Weight ☐☐☐ . ☐ Kg

3.2 Height ☐ . ☐☐ m

3.3 Smoking status ☐ Current ☐ Past ☐ Never

Section 4. Blood Pressure

4.1 Blood pressure ☐☐☐ / ☐☐☐ mmHg
(most recent, measured after 5 mins sitting)

4.2 Anti-hypertensive treatment ☐ No ☐ Yes

if YES → 4.2.1 Select from below:

☐ ACE Inhibitor ☐ A2 Antagonist ☐ Beta Blocker
☐ Calcium Antagonist ☐ Thiazides ☐ Other

Section 5. Diabetic Eye Disease - last 12 months

	No	Yes
5.1 Attended optometrist	☐	☐
5.2 Referred to ophthalmologist	☐	☐
5.3 Attended ophthalmologist	☐	☐
5.4 Fundus examination	☐	☐
5.5 Retinopathy	☐	☐
5.6 Laser treatment	☐	☐
5.7 Right cataract	☐	☐
5.8 Left cataract	☐	☐

Section 6. Diabetic Foot Problems

	No	Yes
6.1 Seen by podiatrist in the last 12 months	☐	☐
6.2 Peripheral neuropathy	☐	☐
6.3 Past History of ulceration	☐	☐
6.4 Foot deformity	☐	☐
6.5 Peripheral vascular disease	☐	☐
6.6 Current foot ulcer	☐	☐

Section 7. Medications & Lipids (most recent results from the last 12 months)

	No	Yes	Contraindicated
7.1 Aspirin	☐	☐	☐
7.2 Other anti-platelets	☐	☐	☐
7.3 Anti-coagulants	☐	☐	☐
7.4 Lipid lowering Rx	☐	☐	
if YES → 7.4.1 Statin	☐	☐	☐
7.4.2 Fibrate	☐	☐	
7.4.3 Ezetrol	☐	☐	
7.4.4 Fish oil	☐	☐	

7.5 Lipids measured ☐ No ☐ Yes

if YES → ☐ Not available

OR

Complete below:

7.5.1 Cholesterol ☐☐ . ☐ mmol/L

7.5.2 LDL ☐ . ☐☐ mmol/L

7.5.3 HDL ☐ . ☐☐ mmol/L

7.5.4 Triglycerides ☐☐ . ☐ mmol/L

7.5.5 Were the above fasting lipids? ☐ No ☐ Yes

Section 8. Complications/Events/Comorbidities

	Last 12 months No	Last 12 months Yes	Previous No	Previous Yes
8.1 Cerebral stroke	☐	☐	☐	☐
8.2 Myocardial infarction	☐	☐	☐	☐
8.3 CABG/Angioplasty	☐	☐	☐	☐
8.4 Congestive cardiac failure	☐	☐	☐	☐
8.5 Lower limb amputation	☐	☐	☐	☐
8.6 End stage kidney disease	☐	☐	☐	☐
8.7 Blindness	☐	☐	☐	☐
8.8 Severe hypoglycaemia	☐	☐	NA	
8.9 Erectile dysfunction	☐	☐	☐	☐
8.10 Dementia	☐	☐	☐	☐

8.11 Malignancy (exclude non-melanotic skin cancers) ☐ Metastatic solid tumour ☐ Leukaemia ☐ Non-metastatic solid tumour ☐ Lymphoma ☐ Not Applicable

8.12 Liver disease ☐ Mild ☐ Moderate/Severe ☐ Not Applicable

Section 9. Renal Function & Blood Glucose Control

9.1 Microalbumin/Proteinuria collected ☐ No ☐ Yes

if YES → 9.1.1 Result ☐☐☐☐ . ☐☐

9.1.2 Units ☐ mg/L ☐ µg/min ☐ mg/24 hr ☐ ratio

9.2 Serum creatinine ☐☐☐☐ µmol/L

9.3.1 HbA1c Result ☐☐ . ☐ % **OR** 9.3.2 ☐☐☐ mmol/mol
(most recent in last 12 months)
