# Supplementary material for: Burden of cardiovascular risk factors and disease among patients with type 1 diabetes: results of the Australian National Diabetes Audit (ANDA)
Source: Cardiovasc Diabetol. 2018 Jun 2;17:77. doi: 10.1186/s12933-018-0726-8 (PMC5984751; doi:10.1186/s12933-018-0726-8)
Supplement: Supplementary file 2 — Additional file 2. ‘ANDA-AQCA 2015 Data Definitions’ outlines the definitions used by healthcare professionals who completed the questionnaire. [file 12933_2018_726_MOESM2_ESM.pdf]

ANDA- AQCA 2015 DATA DEFINITIONS

**Section 1. Patient Demographics**	
Medical Record No.	(Compulsory field). Enter identifier such as record number **or** the first 2 letters of the first name and surname and month and year of birth (e.g. FFSSMMYY) to enable you to check your records if there is a query regarding the data.
Centre ID	Site Identifier.
Site Staff Identifier	Site staff ID.
Date of birth	Record as **DD/MM/YYYY**. [If unknown other than year : Record as 01/01/YYYY].
Sex	Mark **Male** or **Female** indicating phenotypic (physical) sex at birth.
Currently pregnant	If Sex is female, mark **Yes** or **No** if the patient is currently pregnant.
Date of visit	Record the date the patient attended as **DD/MM/2015**.
Initial visit	Mark **No** or **Yes** indicating if this is an initial visit assessment.
Aboriginal/Torres Straits Islander	Mark **No** or **Yes** indicating Aboriginal / Torres Strait Islander background.
Country of birth	Enter the patient's country of birth
NDSS member	Record **No** or **Yes** if a member of the NDSS.
DVA patient	Eligible people whose medical care charges are met by the Department of Veterans' Affairs (DVA).

**Section 2. Diabetes Type & Management**	
Date of diagnosis	Record as **MM/YYYY** of first diagnostic blood glucose estimation. [If date unknown other than year, record as 01/YYYY].
Type of diabetes	Mark **Type1** [IDDM] or **Type2** [NIDDM] or **GDM** or **Don't know**, or **Other** to indicate the clinical classification of diabetes.
Management method	If multiple, **tick all that apply** for management method.
Insulin number of years	If the patient is on Insulin, record the number of years the patient has been on insulin.
Mode of insulin	If the patient is on Insulin, record mode of administration.

**Section 3. Height, Weight & Smoking Status**	
Weight	Record in **kilograms** the weight measurement without shoes or jacket.
Height	Record in **metres** the height measurement without shoes.
Smoking status	Mark **Current** or **Past** or **Never** to indicate smoking activity of any tobacco material.<br>*Current = regular smoking over the past 3months, Past = no regular smoking for 1month or more, Never = never smoked*

**Section 4. Blood Pressure**	
Blood pressure	Record Systolic / Diastolic (mm Hg) measured after ***5 minutes sitting, [1st and 5th phases]***.
Anti-hypertensive treatment	Mark **No** or **Yes** to indicate if the patient is on treatment for hypertension. If YES, select the medication/s from the list
Anti-hypertensive medications	Select the anti-hypertensive medication/s that the patient is currently taking. **If on combination tablet, tick all that apply.**

**Section 5. Diabetic Eye Disease**	
Attended optometrist	Mark **No** or **Yes** to indicate if the patient attended an optometrist in the last 12months.
Referred to ophthalmologist	Mark **No** or **Yes** to indicate if the patient was referred to an ophthalmologist in the last 12months.
Attended ophthalmologist	Mark **No** or **Yes** to indicate if the patient attended an ophthalmologist in the last 12months.
Fundus examination	Mark **No** or **Yes** to indicate if the patient has had an ophthalmological assessment (Direct or Indirect) in the last 12months.
Retinopathy	Mark **No** or **Yes** to indicate if the ophthalmological assessment revealed any diabetic retinopathy.
Laser treatment	Mark **No** or **Yes** to indicate if the patient has had eye laser treatment.
Right & left cataract	Mark **No** or **Yes** to indicate if the patient currently has a cataract or has had one removed previously. Record for **both** eyes.

**Section 6. Diabetic Foot Problems**	
Attended a podiatrist	Mark **No** or **Yes** to indicate if the patient saw a podiatrist.
Peripheral neuropathy	Mark **No** or **Yes** to indicate clinical judgement following assessment using pin prick and vibration or monofilament.
Past history of ulceration	Mark **No** or **Yes** to indicate past history of foot ulceration
Foot deformity	Mark **No** or **Yes** to indicate the presence of **any** foot deformity *(eg. Hallux, hammer or claw toe, flat or high arch, Charcot's)*
Peripheral vascular disease	Mark **No** or **Yes** to indicate peripheral vascular disease. YES = absence of both dorsalis pedis **and** posterior tibial pulses in either foot.
Current foot ulcer	Mark **No** or **Yes** to indicate a current foot ulcer.

**Section 7. Medications & Lipids**	
Aspirin	Mark **No** or **Yes** to indicate whether the patient is on Aspirin. Indicate whether contraindicated.
Other anti-platelets	Mark **No** or **Yes** to indicate whether the patient is on any other anti-platelet treatment (e.g. clopidogrel)
Anti-coagulants	Mark **No** or **Yes** to indicate whether the patient is on anti-coagulant treatment (e.g. Warfarin, novel anti-coagulants)
Lipid lowering treatment	Mark **No** or **Yes** to indicate whether the patient is on lipid lowering treatment. **If Yes**, indicate whether they are on Statin, Fibrate, Ezetrol and/or Fish Oil. Record if contraindicated to statin. **If on combination tablet, tick all that apply.**
Lipids measured	Mark **No** or **Yes** to indicate if lipids have been measured in the past 12 months**. If Yes**, indicate if results are unavailable.
Cholesterol, LDL, HDL, Triglycerides	Record **absolute result** of most recent result of *total, LDL & HDL cholesterol* and *triglycerides* in the last 12months.
Above measured in fasting specimen	Mark **No** or **Yes** to indicate if the lipids reported at items 7.5.1 to 7.5.4 were measured in a fasting specimen.

**Section 8. Complications/Events/Co-morbidities**	
Mark **No** or **Yes** to indicate a history of complication or an event in the last 12months **AND/OR** previously. Answer all.	
Cerebral stroke	Due to vascular disease including TIA.
Myocardial infarction	Evidenced by ECG changes, plasma enzyme changes or medical documentation.
CABG/Angioplasty	CABG, Angioplasty or Stent.
Congestive cardiac failure	Symptomatic congestive cardiac failure with response to specific therapy.
Lower limb amputation	Amputation of toe, forefoot or leg [above or below knee], not due to trauma or causes other than vascular disease.
End stage kidney disease	Requiring dialysis or having undergone kidney transplantation.
Blindness	Patient became legally blind (>6/60) in either eye.
Severe hypoglycaemia	Severe hypoglycaemia requiring assistance of another person to actively administer carbohydrates, glucagon, or other corrective actions.
Erectile dysfunction	History or treatment of failure to achieve or maintain erection sufficient for penetration.
Dementia	Chronic cognitive deficit diagnosed by a clinician.
Malignancy	Indicate type of malignancy **or** if not applicable. *Exclude non-melanotic skin cancers.*
Liver disease	Indicate severity of liver disease **or** if not applicable.<br>***Mild*** *= cirrhosis* ***without*** *portal hypertension, chronic hepatitis,* ***Moderate to severe*** *= cirrhosis* ***with*** *portal hypertension*

**Section 9. Renal Function & Blood Glucose Control**	
Microalbumin/Proteinuria collected	Mark **No** or **Yes** to indicate if microalbumin / proteinuria was done.
Microalbumin/Proteinuria result	Record absolute amount of albumin [**mg/L**] or as albumin excretion rate [AER: **µg/min** or **mg/24hr**] or **Ratio**.
Microalbumin/Proteinuria units	Mark the applicable units.
Serum creatinine	Record **absolute result** measurement of serum creatinine in **MICROMOLS/L** [µmol/L].
HbA1c result	Record **absolute result** [%] or mmol/mol of the most recent HbA1c result in the last 12months.
