# Supplementary material for: Burden of cardiovascular risk factors and disease among patients with type 1 diabetes: results of the Australian National Diabetes Audit (ANDA)
Source: Cardiovasc Diabetol. 2018 Jun 2;17:77. doi: 10.1186/s12933-018-0726-8 (PMC5984751; doi:10.1186/s12933-018-0726-8)
Supplement: Supplementary file 3 — Additional file 3: Table S1. Multiple imputation for cardiovascular outcomes of interest. [file 12933_2018_726_MOESM3_ESM.docx]

| **Table S1. Multiple imputation for cardiovascular outcomes of interest** | | | | | |
| --- | --- | --- | --- | --- | --- |
| **Cardiovascular Disease (composite)** | | | | | |
| **Variables** | Non-imputed Analyses | | Imputed Analyses | |  |
|  | OR (95% CI) | P-Value | OR (95% CI) | P-Value | Missing data  n (%) |
| Female Sex | 0.90 (0.46-1.78) | 0.764 | NA |  | NA |
| Age (years) | 1.06 (1.03-1.09) | <0.001 | NA |  | NA |
| Diabetes duration group | 1.05 (1.01-1.10) | 0.018 | 1.08 (1.06-1.11) | <0.001 | 16 (1.4) |
| HbA1c (%) |  |  |  |  |  |
| HDL-Cholesterol (mmol/L) | 0.43 (0.21-0.90) | 0.025 | 0.43 (0.26-0.71) | 0.001 | 518 (45.3) |
| Systolic BP (mmHg) |  |  |  |  |  |
| Diastolic BP (mmHg) | 0.96 (0.93-1.00) | 0.048 | 0.97 (0.95-0.99) | 0.002 | 58 (5.1) |
| BMI Categories |  |  |  |  |  |
| Ever smoked | 2.40 (1.26-4.58) | 0.008 | 3.05 (1.99-4.67) | <0.001 | 127 (11.1) |
| Albuminuria |  |  |  |  |  |
| eGFR (mL/min/1.73m^2^) |  |  |  |  |  |
| Antihypertensive Rx | 2.44 (1.15-5.18) | 0.020 | 5.11 (3.33-7.84) | <0.001 | 29 (2.5) |
| Lipid Lowering Rx |  |  |  |  |  |
| Retinopathy |  |  |  |  |  |
| **Stroke** | | | | | |
| **Variables** | Non-imputed Analyses | | Imputed Analyses | |  |
|  | OR (95% CI) | P-Value | OR (95% CI) | P-Value | Missing data  n (%) |
| Female Sex | 0.49 (0.16-1.47) | 0.201 | NA |  | NA |
| Age (years) | 1.05 (1.01-1.08) | 0.006 | NA |  | NA |
| Diabetes duration group |  |  |  |  |  |
| HbA1c (%) |  |  |  |  |  |
| HDL-Cholesterol (mmol/L) |  |  |  |  |  |
| Systolic BP (mmHg) |  |  |  |  |  |
| Diastolic BP (mmHg) |  |  |  |  |  |
| BMI Categories |  |  |  |  |  |
| Ever smoked |  |  |  |  |  |
| Albuminuria |  |  |  |  |  |
| eGFR (mL/min/1.73m^2^) | 0.98 (0.96-1.00) | 0.030 | 0.98 (0.97-0.99) | <0.001 | 201 (17.6) |
| Antihypertensive Rx |  |  |  |  |  |
| Lipid Lowering Rx |  |  |  |  |  |
| Retinopathy |  |  |  |  |  |
| **Myocardial Infarction** | | | | | |
| **Variables** | Non-imputed Analyses | | Imputed Analyses | |  |
|  | OR (95% CI) | P-Value | OR (95% CI) | P-Value | Missing data  n (%) |
| Female Sex | 0.97 (0.39-2.41) | 0.943 | NA |  | NA |
| Age (years) | 1.09 (1.05-1.13) | <0.001 | NA |  | NA |
| Diabetes duration group |  |  |  |  |  |
| HbA1c (%) |  |  |  |  |  |
| HDL-Cholesterol (mmol/L) | 0.20 (0.06-0.68) | 0.010 | 0.35 (0.15-0.83) | 0.018 | 518 (45.3) |
| Systolic BP (mmHg) |  |  |  |  |  |
| Diastolic BP (mmHg) |  |  |  |  |  |
| BMI Categories |  |  |  |  |  |
| Ever smoked |  |  |  |  |  |
| Albuminuria |  |  |  |  |  |
| eGFR (mL/min/1.73m^2^) |  |  |  |  |  |
| Antihypertensive Rx | 5.06 (1.38-18.54) | 0.014 | 15.91 (7.65-33.12) | <0.001 | 29 (2.5) |
| Lipid Lowering Rx |  |  |  |  |  |
| Retinopathy |  |  |  |  |  |
| **Coronary Artery Bypass Graft / Angioplasty** | | | | | |
| **Variables** | Non-imputed Analyses | | Imputed Analyses | |  |
|  | OR (95% CI) | P-Value | OR (95% CI) | P-Value | Missing data  n (%) |
| Female Sex | 0.93 (0.34-2.52) | 0.884 | NA |  | NA |
| Age (years) | 1.08 (1.03-1.13) | 0.001 | NA |  | NA |
| Diabetes duration group |  |  |  |  |  |
| HbA1c (%) |  |  |  |  |  |
| HDL-Cholesterol (mmol/L) | 0.23 (0.06-0.92) | 0.038 | 0.45 (0.19-1.08) | 0.072 | 518 (45.3) |
| Systolic BP (mmHg) |  |  |  |  |  |
| Diastolic BP (mmHg) |  |  |  |  |  |
| BMI Categories |  |  |  |  |  |
| Ever smoked |  |  |  |  |  |
| Albuminuria |  |  |  |  |  |
| eGFR (mL/min/1.73m^2^) |  |  |  |  |  |
| Antihypertensive Rx | 8.96 (1.12-71.54) | 0.039 | 21.90 (9.79-48.99) | <0.001 | 29 (2.5) |
| Lipid Lowering Rx |  |  |  |  |  |
| Retinopathy |  |  |  |  |  |
| **Peripheral Vascular Disease** | | | | | |
| **Variables** | Non-imputed Analyses | | Imputed Analyses | |  |
|  | OR (95% CI) | P-Value | OR (95% CI) | P-Value | Missing data  n (%) |
| Female Sex | 1.08 (0.49-2.39) | 0.851 | NA |  | NA |
| Age (years) | 1.04 (1.01-1.07) | 0.005 | NA |  | NA |
| Diabetes duration group |  |  |  |  |  |
| HbA1c (%) |  |  |  |  |  |
| HDL-Cholesterol (mmol/L) |  |  |  |  |  |
| Systolic BP (mmHg) |  |  |  |  |  |
| Diastolic BP (mmHg) |  |  |  |  |  |
| BMI Categories |  |  |  |  |  |
| Ever smoked |  |  |  |  |  |
| Albuminuria |  |  |  |  |  |
| eGFR (mL/min/1.73m^2^) | 0.97 (0.96-0.99) | 0.002 | 0.97 (0.96-0.98) | <0.001 | 201 (17.6) |
| Antihypertensive Rx |  |  |  |  |  |
| Lipid Lowering Rx |  |  |  |  |  |
| Retinopathy | 2.47 (1.06-5.74) | 0.036 | 2.79 (1.65-4.72) | <0.001 | 18 (1.6) |
| **Congestive Cardiac Failure** | | | | | |
| **Variables** | Non-imputed Analyses | | Imputed Analyses | |  |
|  | OR (95% CI) | P-Value | OR (95% CI) | P-Value | Missing data  n (%) |
| Female Sex | 1.51 (0.30-7.47) | 0.614 | NA |  | NA |
| Age (years) | 1.15 (1.05-1.25) | 0.002 | NA |  | NA |
| Diabetes duration group |  |  |  |  |  |
| HbA1c (%) |  |  |  |  |  |
| HDL-Cholesterol (mmol/L) |  |  |  |  |  |
| Systolic BP (mmHg) |  |  |  |  |  |
| Diastolic BP (mmHg) |  |  |  |  |  |
| BMI Categories |  |  |  |  |  |
| Ever smoked |  |  |  |  |  |
| Albuminuria |  |  |  |  |  |
| eGFR (mL/min/1.73m^2^) |  |  |  |  |  |
| Antihypertensive Rx |  |  |  |  |  |
| Lipid Lowering Rx |  |  |  |  |  |
| Retinopathy |  |  |  |  |  |
| Rx: treatment, NA: not applicable  Cardiovascular outcomes of interest (missing observation count): CVD (8), stroke (4), MI (4), CABG/angioplasty (4), PVD (8) and CCF (0). A multivariate normal distribution was used to impute the missing values, with 10 simulated datasets and random seed number set at 12345. Sex and age were used as predictor variables. | | | | | |
