# Supplementary material for: Burden of cardiovascular risk factors and disease among patients with type 1 diabetes: results of the Australian National Diabetes Audit (ANDA)
Source: Cardiovasc Diabetol. 2018 Jun 2;17:77. doi: 10.1186/s12933-018-0726-8 (PMC5984751; doi:10.1186/s12933-018-0726-8)
Supplement: Supplementary file 4 — Additional file 4: Table S2. Missing data for cardiovascular risk factors and outcomes of interest. [file 12933_2018_726_MOESM4_ESM.docx]

| **Table S2. Missing data for cardiovascular risk factors and outcomes of interest** | | |
| --- | --- | --- |
| **Variables** | Missing data  n (%) |  |
| Cardiovascular disease | 8 (0.7) |  |
| Stroke | 4 (0.3) |  |
| Myocardial infarction | 4 (0.3) |  |
| Coronary artery bypass graft/angioplasty | 4 (0.3) |  |
| Peripheral vascular disease | 8 (0.7) |  |
| Congestive cardiac failure | 0 (0.0) |  |
| Sex | 26 (2.2) |  |
| Age (years) | 0 (0.0) |  |
| Diabetes duration (years) | 17 (1.5) |  |
| Diabetes duration (>20.0years) | 17 (1.5) |  |
| HbA1c (%) | 143 (12.2) |  |
| High density lipoprotein-cholesterol^#^ | 533 (45.6) |  |
| Low density lipoprotein-cholesterol^#^ | 590 (50.5) |  |
| Total cholesterol^#^ | 412 (35.2) |  |
| Triglycerides^#^ | 440 (37.6) |  |
| Systolic blood pressure^^^ | 66 (5.7) |  |
| Diastolic blood pressure^^^ | 66 (5.7) |  |
| Body mass index categories | 162 (13.9) |  |
| Ever smoked | 138 (11.8) |  |
| Albuminuria | 462 (39.5) |  |
| Estimated glomerular filtration rate^*^ | 227 (19.4) |  |
| Antihypertensive Rx | 30 (2.6) |  |
| Lipid Lowering Rx | 16 (1.4) |  |
| Retinopathy | 18 (1.5) |  |
| ^#^: mmol/L, ^^^: mmHg, ^*^: mL/min/1.73m^2^, Rx: Treatment | |  |
