# Supplementary material for: Burden of cardiovascular risk factors and disease among patients with type 1 diabetes: results of the Australian National Diabetes Audit (ANDA)
Source: Cardiovasc Diabetol. 2018 Jun 2;17:77. doi: 10.1186/s12933-018-0726-8 (PMC5984751; doi:10.1186/s12933-018-0726-8)
Supplement: Supplementary file 5 — Additional file 5: Table S3. a Distribution of variables for cardiovascular outcomes of interest and b Distribution of variables for congestive cardiac failure. [file 12933_2018_726_MOESM5_ESM.docx]

| **Table S3a. Distribution of risk factors for cardiovascular outcomes of interest** | | | | | | |  |  |
| --- | --- | --- | --- | --- | --- | --- | --- | --- |
| **Variables** | CABG / Angioplasty  N = 57 | No CABG / Angioplasty  N = 1108 | PVD  N = 80 | No PVD  N = 1081 | Stroke  N = 32 | No Stroke  N = 1133 | MI  N = 55 | No MI  N = 1110 |
| Female Sex, n (%) | 18 (31.6%) | 590 (54.5%) | 37 (46.3%) | 570 (54.0%) | 9 (28.1%) | 599 (54.1%) | 18 (32.7%) | 590 (54.4%) |
| Age (years), mean (±SD) | 63.8 (±11.3) | 38.8 (±16.1) | 58.6 (±14.7) | 38.6 (±16.1) | 58.4 (±16.6) | 39.5 (±16.5) | 62.0 (±12.7) | 38.9 (±16.2) |
| Age (years), median (IQR) | 65.1 (56.3-72.0) | 35.8 (24.7-49.8) | 57.6 (48.9-69.1) | 35.2 (24.6-49.2) | 58.6 (48.7-70.5) | 36.5 (24.8-51.2) | 64.4 (53.0-72.0) | 36.0 (24.7-50.2) |
| Diabetes duration (years), mean (±SD) | 41.5 (±14.2) | 18.1 (±13.4) | 33.5 (±14.2) | 18.3 (±13.8) | 32.9 (±16.1) | 18.9 (±14.2) | 36.9 (±15.6) | 18.4 (±13.8) |
| Diabetes duration (years), median (IQR) | 42.0 (31.0-51.0) | 15.0 (8.0-26.0) | 34.0 (24.0-44.0) | 15.0 (8.0-26.0) | 36.5 (20.5-44.5) | 16.0 (8.0-27.0) | 38.5 (26.0-47.0) | 16.0 (8.0-26.0) |
| Diabetes duration (>20years), n (%) | 55 (96.5%) | 420 (38.5%) | 65 (84.4%) | 410 (38.4%) | 24 (75.0%) | 451 (40.4%) | 47 (87.0%) | 428 (39.1%) |
| HbA1c (%), mean (±SD) | 8.4 (±1.4) | 8.5 (±1.9) | 8.8 (±1.4) | 8.5 (±1.8) | 9.1 (±1.6) | 8.5 (±1.8) | 8.4 (±1.3) | 8.5 (±1.9) |
| HDL-C^#^, mean (±SD) | 1.30 (±0.38) | 1.55 (±0.55) | 1.35 (±0.48) | 1.55 (±0.55) | 1.29 (±0.31) | 1.54 (±0.55) | 1.29 (±0.30) | 1.55 (±0.55) |
| LDL-C^#^, mean (±SD) | 1.99 (±0.55) | 2.59 (±0.96) | 2.19 (±0.85) | 2.58 (±0.95) | 2.07 (±0.80) | 2.57 (±0.95) | 2.14 (±0.92) | 2.58 (±0.95) |
| Total-C^#^, mean (±SD) | 3.86 (±0.76) | 4.78 (±1.08) | 4.24 (±1.21) | 4.77 (±1.07) | 4.14 (±1.10) | 4.75 (±1.08) | 4.00 (±0.96) | 4.77 (±1.08) |
| Triglycerides^#^, mean (±SD) | 1.22 (±0.77) | 1.38 (±1.45) | 1.68 (±1.88) | 1.34 (±1.38) | 1.98 (±2.40) | 1.35 (±1.37) | 1.15 (±0.71) | 1.38 (±1.45) |
| Systolic BP^^^, mean (±SD) | 133 (±20) | 124 (±17) | 131 (±22) | 124 (±17) | 131 (±17) | 124 (±17) | 134 (±21) | 124 (±17) |
| Diastolic BP^^^, mean (±SD) | 72 (±9) | 74 (±10) | 71 (±10) | 74 (±10) | 74 (±10) | 74 (±10) | 74 (±10) | 74 (±10) |
| BMI Categories, n (%)   - <18.5 kg/m^2^ - 18.5 - <25 kg/m^2^ - 25 - <30 kg/m^2^ - >30 kg/m^2^ | 0 (0.0%)  19 (40.4%)  13 (27.7%)  15 (31.9%) | 20 (2.1%)  397 (41.5%)  302 (31.6%)  237 (24.8%) | 3 (4.5%)  26 (38.8%)  19 (28.4%)  19 (28.4%) | 16 (1.7%)  391 (41.9%)  294 (31.5%)  233 (25.0%) | 0 (0.0%)  11 (37.9%)  11 (37.9%)  7 (24.1%) | 20 (2.1%)  405 (41.6%)  304 (31.2%)  245 (25.2%) | 1 (2.1%)  19 (40.4%)  14 (29.8%)  13 (27.7%) | 19 (2.0%)  397 (41.5%)  301 (31.5%)  239 (25.0%) |
| Ever smoked, n (%) | 30 (56.6%) | 366 (37.5%) | 50 (67.6%) | 345 (36.2%) | 17 (58.6%) | 379 (37.9%) | 29 (58.0%) | 367 (37.5%) |
| Albuminuria, n (%) | 16 (44.4%) | 204 (30.5%) | 27 (55.1%) | 192 (29.3%) | 11 (50.0%) | 209 (30.6%) | 19 (48.7%) | 201 (30.2%) |
| eGFR^*^, mean (±SD) | 68 (±25) | 99 (±27) | 64 (±31) | 100 (±26) | 73 (±31) | 98 (±27) | 66 (±26) | 99 (±27) |
| Antihypertensive Rx,  n (%) | 51 (92.7%) | 268 (24.8%) | 50 (64.1%) | 267 (25.3%) | 23 (74.2%) | 296 (26.8%) | 48 (88.9%) | 271 (25.1%) |
| Lipid Lowering Rx, n (%) | 52 (92.9%) | 290 (26.5%) | 52 (65.8%) | 288 (27.0%) | 24 (75.0%) | 318 (28.5%) | 47 (87.0%) | 295 (26.9%) |
| Retinopathy, n (%) | 32 (57.1%) | 252 (23.1%) | 50 (64.1%) | 233 (21.8%) | 17 (54.8%) | 267 (23.9%) | 29 (52.7%) | 255 (23.3%) |
| ^#^: mmol/L, ^^^: mmHg, ^*^: mL/min/1.73m^2^, Rx: Treatment | | | | | | | | |

| **Table S3b. Distribution of variables for congestive cardiac failure** | | |
| --- | --- | --- |
| **Variables** | CCF  N = 15 | No CCF  N = 1154 |
| Female Sex, n (%) | 8 (53.3%) | 601 (53.3%) |
| Age (years), mean (±SD) | 67.2 (±12.2) | 39.6 (±16.5) |
| Age (years), median (IQR) | 66.3 (59.9-74.7) | 36.6 (24.9-51.6) |
| Diabetes duration (years), mean (±SD) | 40.5 (±13.9) | 19.0 (±14.2) |
| Diabetes duration (years), median (IQR) | 42.0 (33.0-45.0) | 16.0 (8.0-27.0) |
| Diabetes duration (>20years), n (%) | 14 (93.3%) | 462 (40.6%) |
| HbA1c (%), mean (±SD) | 8.4 (±1.5) | 8.5 (±1.8) |
| HDL-C^#^, mean (±SD) | 1.80 (±1.83) | 1.53 (±0.50) |
| LDL-C^#^, mean (±SD) | 2.55 (±2.10) | 2.55 (±0.92) |
| Total-C^#^, mean (±SD) | 4.29 (±1.88) | 4.74 (±1.07) |
| Triglycerides^#^, mean (±SD) | 3.07 (±4.10) | 1.34 (±1.33) |
| Systolic BP^^^, mean (±SD) | 130 (±18) | 124 (±17) |
| Diastolic BP^^^, mean (±SD) | 70 (±11) | 74 (±10) |
| BMI Categories, n (%)   - <18.5 kg/m^2^ - 18.5 - <25 kg/m^2^ - 25 - <30 kg/m^2^ - >30 kg/m^2^ | 0 (0.0%)  0 (0.0%)  8 (57.1%)  6 (42.9%) | 20 (2.0%)  419 (42.2%)  307 (30.9%)  247 (24.9%) |
| Ever smoked, n (%) | 7 (46.7%) | 390 (38.4%) |
| Albuminuria, n (%) | 7 (70.0%) | 213 (30.6%) |
| eGFR^*^, mean (±SD) | 47 (±22) | 98 (±27) |
| Antihypertensive Rx, n (%) | 14 (93.3%) | 306 (27.2%) |
| Lipid Lowering Rx, n (%) | 11 (73.3%) | 331 (29.1%) |
| Retinopathy, n (%) | 13 (86.7%) | 272 (23.9%) |
| ^#^: mmol/L, ^^^: mmHg, ^*^: mL/min/1.73m^2^, Rx: Treatment | | |
