# Supplementary material for: Burden of cardiovascular risk factors and disease among patients with type 1 diabetes: results of the Australian National Diabetes Audit (ANDA)
Source: Cardiovasc Diabetol. 2018 Jun 2;17:77. doi: 10.1186/s12933-018-0726-8 (PMC5984751; doi:10.1186/s12933-018-0726-8)
Supplement: Supplementary file 6 — Additional file 6: Table S4. Population attributable risk for cardiovascular outcomes of interest. [file 12933_2018_726_MOESM6_ESM.docx]

| **Table S4. Population attributable risk for cardiovascular outcomes of interest** | | | | | |
| --- | --- | --- | --- | --- | --- |
| **Cardiovascular Disease (composite)** | | | | | |
| **Variables** | Multivariable Analyses | | Population Attributable Risk (PAR) | | |
|  | OR (95% CI) | P-Value | PAR% (95% CI) P Value | | |
| Female Sex | 0.90 (0.46-1.78) | 0.764 |  |  |  |
| Age (years) | 1.06 (1.03-1.09) | <0.001 |  |  |  |
| Diabetes duration group | 1.05 (1.01-1.10) | 0.018 | 6.5 (1.4-11.6) 0.012 | | |
| HbA1c (%) |  |  |  |  |  |
| HDL-Cholesterol (mmol/L) | 0.43 (0.21-0.90) | 0.025 |  |  |  |
| Systolic BP (mmHg) |  |  |  |  |  |
| Diastolic BP (mmHg) | 0.96 (0.93-1.00) | 0.048 |  |  |  |
| BMI Categories |  |  |  |  |  |
| Ever smoked | 2.40 (1.26-4.58) | 0.008 | 3.9 (1.0-6.7) 0.008 | | |
| Albuminuria |  |  |  |  |  |
| eGFR (mL/min/1.73m^2^) |  |  |  |  |  |
| Antihypertensive Rx | 2.44 (1.15-5.18) | 0.020 | 5.1 (0.9-9.3) 0.018 | | |
| Lipid Lowering Rx |  |  |  |  |  |
| Retinopathy |  |  |  |  |  |
| **Stroke** | | | | | |
| **Variables** | Multivariable Analyses | | Population Attributable Risk (PAR) | | |
|  | OR (95% CI) | P-Value | PAR% (95% CI) P Value | | |
| Female Sex | 0.49 (0.16-1.47) | 0.201 |  |  |  |
| Age (years) | 1.05 (1.01-1.08) | 0.006 |  |  |  |
| Diabetes duration group |  |  |  |  |  |
| HbA1c (%) |  |  |  |  |  |
| HDL-Cholesterol (mmol/L) |  |  |  |  |  |
| Systolic BP (mmHg) |  |  |  |  |  |
| Diastolic BP (mmHg) |  |  |  |  |  |
| BMI Categories |  |  |  |  |  |
| Ever smoked |  |  |  |  |  |
| Albuminuria |  |  |  |  |  |
| eGFR (mL/min/1.73m^2^) | 0.98 (0.96-1.00) | 0.030 |  |  |  |
| Antihypertensive Rx |  |  |  |  |  |
| Lipid Lowering Rx |  |  |  |  |  |
| Retinopathy |  |  |  |  |  |
| **Myocardial Infarction** | | | | | |
| **Variables** | Multivariable Analyses | | Population Attributable Risk (PAR) | | |
|  | OR (95% CI) | P-Value | PAR% (95% CI) P Value | | |
| Female Sex | 0.97 (0.39-2.41) | 0.943 |  |  |  |
| Age (years) | 1.09 (1.05-1.13) | <0.001 |  |  |  |
| Diabetes duration group |  |  |  |  |  |
| HbA1c (%) |  |  |  |  |  |
| HDL-Cholesterol (mmol/L) | 0.20 (0.06-0.68) | 0.010 |  |  |  |
| Systolic BP (mmHg) |  |  |  |  |  |
| Diastolic BP (mmHg) |  |  |  |  |  |
| BMI Categories |  |  |  |  |  |
| Ever smoked |  |  |  |  |  |
| Albuminuria |  |  |  |  |  |
| eGFR (mL/min/1.73m^2^) |  |  |  |  |  |
| Antihypertensive Rx | 5.06 (1.38-18.54) | 0.014 | 4.8 (1.8-7.8) 0.002 | | |
| Lipid Lowering Rx |  |  |  |  |  |
| Retinopathy |  |  |  |  |  |
| **Coronary Artery Bypass Graft / Angioplasty** | | | | | |
| **Variables** | Multivariable Analyses | | Population Attributable Risk (PAR) | | |
|  | OR (95% CI) | P-Value | PAR% (95% CI) P Value | | |
| Female Sex | 0.93 (0.34-2.52) | 0.884 |  |  |  |
| Age (years) | 1.08 (1.03-1.13) | 0.001 |  |  |  |
| Diabetes duration group |  |  |  |  |  |
| HbA1c (%) |  |  |  |  |  |
| HDL-Cholesterol (mmol/L) | 0.23 (0.06-0.92) | 0.038 |  |  |  |
| Systolic BP (mmHg) |  |  |  |  |  |
| Diastolic BP (mmHg) |  |  |  |  |  |
| BMI Categories |  |  |  |  |  |
| Ever smoked |  |  |  |  |  |
| Albuminuria |  |  |  |  |  |
| eGFR (mL/min/1.73m^2^) |  |  |  |  |  |
| Antihypertensive Rx | 8.96 (1.12-71.54) | 0.039 | 11.2 (5.0-17.5) <0.001 | | |
| Lipid Lowering Rx |  |  |  |  |  |
| Retinopathy |  |  |  |  |  |
| **Peripheral Vascular Disease** | | | | | |
| **Variables** | Multivariable Analyses | | Population Attributable Risk (PAR) | | |
|  | OR (95% CI) | P-Value | PAR% (95% CI) P Value | | |
| Female Sex | 1.08 (0.49-2.39) | 0.851 |  |  |  |
| Age (years) | 1.04 (1.01-1.07) | 0.005 |  |  |  |
| Diabetes duration group |  |  |  | | |
| HbA1c (%) |  |  |  |  |  |
| HDL-Cholesterol (mmol/L) |  |  |  |  |  |
| Systolic BP (mmHg) |  |  |  |  |  |
| Diastolic BP (mmHg) |  |  |  |  |  |
| BMI Categories |  |  |  |  |  |
| Ever smoked |  |  |  | | |
| Albuminuria |  |  |  |  |  |
| eGFR (mL/min/1.73m^2^) | 0.97 (0.96-0.99) | 0.002 |  |  |  |
| Antihypertensive Rx |  |  |  |  |  |
| Lipid Lowering Rx |  |  |  |  |  |
| Retinopathy | 2.47 (1.06-5.74) | 0.036 | 2.7 (0.2-5.2) |  | 0.035 |
| **Congestive Cardiac Failure** | | | | | |
| **Variables** | Multivariable Analyses | | Population Attributable Risk (PAR) | | |
|  | OR (95% CI) | P-Value | PAR% (95% CI) P Value | | |
| Female Sex | 1.51 (0.30-7.47) | 0.614 |  |  |  |
| Age (years) | 1.15 (1.05-1.25) | 0.002 |  |  |  |
| Diabetes duration group |  |  |  | | |
| HbA1c (%) |  |  |  |  |  |
| HDL-Cholesterol (mmol/L) |  |  |  |  |  |
| Systolic BP (mmHg) |  |  |  |  |  |
| Diastolic BP (mmHg) |  |  |  |  |  |
| BMI Categories |  |  |  |  |  |
| Ever smoked |  |  |  | | |
| Albuminuria |  |  |  |  |  |
| eGFR (mL/min/1.73m^2^) |  |  |  |  |  |
| Antihypertensive Rx |  |  |  |  |  |
| Lipid Lowering Rx |  |  |  |  |  |
| Retinopathy |  |  |  |  |  |
| Rx: treatment  Population attributable risk was calculated for significant categorical variables from the multivariable model. | | | | | |
